# Silencing NKD2 by promoter region hypermethylation promotes gastric cancer invasion and metastasis by up-regulating SOX18 in human gastric cancer

**DOI:** 10.18632/oncotarget.5272

**Published:** 2015-09-14

**Authors:** Yan Jia, Baoping Cao, Yunsheng Yang, Enqiang Linghu, Qimin Zhan, Youyong Lu, Yingyan Yu, James G. Herman, Mingzhou Guo

**Affiliations:** ^1^ Department of Gastroenterology & Hepatology, Chinese PLA General Hospital, Beijing 100853, China; ^2^ Department of Breast Oncology, Tianjin Medical University Cancer Institute and Hospital, National Clinical Research Center for Cancer, and Key Laboratory of Cancer Prevention and Therapy, Tianjin 300060, China; ^3^ Medical College of NanKai University, Tianjin 300071, China; ^4^ State Key Laboratory of Molecular Oncology, Cancer Institute and Hospital, Chinese Academy of Medical Sciences & Peking Union Medical College, Beijing 100021, China; ^5^ Laboratory of Molecular Oncology, Key Laboratory of Carcinogenesis and Translational Research (Ministry of Education), Peking University Cancer Hospital/Institute, Beijing 100142, China; ^6^ Shanghai Ruijin Hospital, Shanghai Jiao Tong University School of Medicine, Shanghai 200240, China; ^7^ The Hillman Cancer Center, University of Pittsburgh Cancer Institute, Pittsburgh, PA 15213, USA

**Keywords:** NKD1, NKD2, SOX18, DNA methylation, gastric cancer

## Abstract

Naked cuticle homolog2 (*NKD2*) is located in chromosome 5p15.3, which is frequently loss of heterozygosity in human colorectal and gastric cancers. In order to understand the mechanism of NKD2 in gastric cancer development, 6 gastric cancer cell lines and 196 cases of human primary gastric cancer samples were involved. Methylation specific PCR (MSP), gene expression array, flow cytometry, transwell assay and xenograft mice model were employed in this study. The expression of *NKD1* and *NKD2* was silenced by promoter region hypermethylation. *NKD1* and *NKD2* were methylated in 11.7% (23/196) and 53.1% (104/196) in human primary gastric cancer samples. *NKD2* methylation is associated with cell differentiation, TNM stage and distant metastasis significantly (all *P* < 0.05), and the overall survival time is longer in *NKD2* unmethylated group compared to *NKD2* methylated group (*P* < 0.05). Restoration of NKD2 expression suppressed cell proliferation, colony formation, cell invasion and migration, induced G2/M phase arrest, and sensitized cancer cells to docetaxel. NKD2 inhibits SOX18 and MMP-2,7,9 expression and suppresses BGC823 cell xenograft growth. In conclusion, NKD2 methylation may serve as a poor prognostic and chemo-sensitive marker in human gastric cancer. NKD2 impedes gastric cancer metastasis by inhibiting SOX18.

## INTRODUCTION

Almost one million new cases of stomach cancer were estimated to have occurred in 2012, making it the fifth most common malignancy in the world. More than 70% of cases occur in developing countries and half of the world's total cases occur in Eastern Asia [1]. Etiologically, gastric cancer is associated with the combined effects of environmental factors and susceptible genetic variants, including the accumulation of genetic and epigenetic alterations [2–4]. *H. pylori* infection, smoking, and ingestion of salt-preserved foods and salt are regarded as risk factors for gastric cancer [5]. Approximately 1–3% of gastric cancer is hereditary diffuse gastric cancer. In roughly 30% of familial gastric cancers, a germline mutation in one allele of the *E-cadherin* gene (*CDH1*) can be identified. Inactivation of the second allele occurs either by mutation or hypermethylation. The estimated life-time risk of developing gastric cancer in carriers of a *CDH1* mutation is 67% in men and 83% in women [5–7]. According to a recent study that performed targeted deep sequencing in 167 cases of gastric cancer, TP53 was among the most commonly mutated genes (35%). Other frequently mutated genes identified were *PI3KCA* (6%), *CTNNB1* (5%), *KRAS* (5%) and *SMAD4* (4%) [8]. A number of tumor suppressor genes, such as *hMLM1, p14, p15, p16, GSTP1, RASSF1, COX-2, APC, CDH1, CDH4, DAP-K, THBS1, TIMP-3, RARβ, MGMT, CHFR, DCC, RUNX3, TSLC1, BCL2L10, IRX1, CMDM* and *UCHL1*, are frequently silenced by hypermethylation in gastric cancer [9–12].

Naked cuticle homolog1 (*NKD1*) and 2 (*NKD2*) are two mammalian orthologs of drosophila naked cuticle [13, 14]. *NKD1* is located in human chromosome 16q12.1, which has frequent loss of heterozygosity in human breast and hepatocellular carcinoma [15–17]. *NKD2* is located in chromosome 5p15.3, and loss of heterozygosity is frequently found in this region in colorectal and gastric cancer [18, 19]. In both zebrafish and mice, NKD inhibits canonical and non-canonical Wnt signaling [14, 20, 21]. Myristoylation of mammalian NKD2, but not NKD1, interacts with the cytoplasmic tail of TGF-α and accelerates TGF-α processing and cell-surface delivery [22]. In addition, overexpression of TGF-α protects the NKD2 protein from rapid ubiquitin-mediated proteasomal degradation in an EGFR-independent manner in HEK293 cells [23]. NKD2 has been reported to suppress tumor growth and metastasis in osteosarcoma [24]. In this study, we focused on the epigenetic changes and mechanisms of NKD2 in human gastric carcinogenesis.

## RESULTS

### NKD1 and NKD2 expression are silenced by promoter region hypermethylation in gastric cancer cell lines

**Figure 1:**
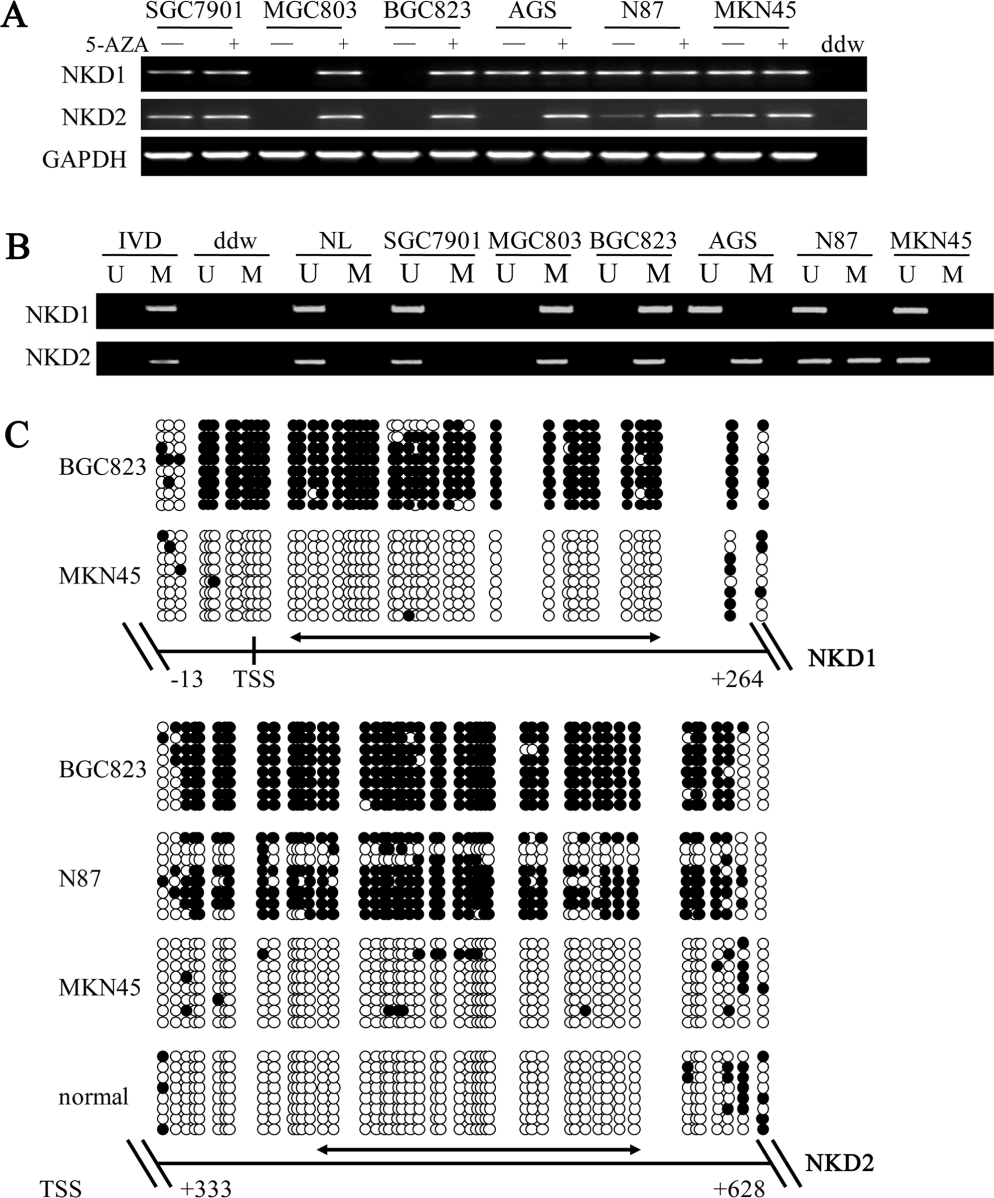
The expression of NKD1 and NKD2 and their methylation status in human gastric cancer cells **A.** Semi-quantitative RT-PCR shows NKD1 and NKD2 expression levels in gastric cancer cell lines. SGC7901, MGC803, BGC823, AGS, N87 and MKN45 are gastric cancer cell lines. 5-AZA: 5-aza-2′-deoxycytidine; GAPDH: internal control of RT-PCR; ddw: double distilled water. (−): absence of 5-AZA; (+): presence of 5-AZA. **B.** MSP results of *NKD1* and *NKD2* in gastric cancer cell lines. U: unmethylated alleles; M: methylated alleles; IVD: *in vitro* methylated DNA, serves as methylation control; NL: normal peripheral lymphocytes DNA, serves as unmethylation control; ddw: double distilled water. **C.** BSSQ results of *NKD1* in BGC823 and MKN45 cells and *NKD2* in BGC823, N87, MKN45 cells and normal gastric mucosa. Upper portion of double-headed arrow: MSP PCR product size was 194 bp in *NKD1* and bisulfite sequencing focused on a 277 bp region of the CpG island (from −13 to +264) around the *NKD1* transcription start site. Lower portion of double-headed arrow: MSP PCR product spanned 166 bp in *NKD2*. Bisulfite sequencing focused on a 295 bp region of the CpG island (+333 bp to +628 bp) downstream of the *NKD2* transcription start site. Filled circles: methylated CpG sites, open circles: unmethylated CpG sites. TSS: transcription start site; normal: normal gastric mucosa.

To explore the regulation mechanisms of the *NKD* gene family in gastric cancer, the expression levels of NKD1 and NKD2 were examined by semi-quantitative RT-PCR. Loss of NKD1 expression was observed in BGC823 and MGC803 cells, and NKD1 expression was found in SGC7901, AGS, N87 and MKN45 cells. Loss of NKD2 expression was found in BGC823, MGC803 and AGS cells, and low level expression of NKD2 was detected in N87 cells. The expression of NKD2 was observed in SGC7901 and MKN45 cells (Figure 1A). Promoter region methylation was detected by methylation-specific PCR (MSP). *NKD1* was completely methylated in BGC823 and MGC803 cells, and it was unmethylated in SGC7901, AGS, N87 and MKN45 cells. *NKD2* was found to be completely methylated in BGC823, MGC803 and AGS cells, partially methylated in N87 cells, and unmethylated in SGC7901 and MKN45 cells (Figure 1B). The above results demonstrate that loss or reduction of NKD expression is correlated with promoter region hypermethylation in human gastric cancer cells. Representative bisulfite sequencing results are shown in Figure 1C. *NKD1* was densely methylated in the promoter region in BGC823 cells and unmethylated in MKN45 cells. *NKD2* was densely methylated in BGC823, partially methylated in N87 and unmethylated in MKN45 cells and normal gastric mucosa. These results further validated the efficiency of the MSP primers and the density of promoter region methylation.

To further determine whether expression levels of the *NKD* genes were regulated by promoter region methylation, we treated cells with 5-aza-2′-deoxycytidine (DAC), a DNA methylation transferase (DNMTs) inhibitor that induces re-expression of methylated genes through de-methylation [25, 26]. Re-expression of NKD1 was induced in BGC823 and MGC803 cells. No NKD1 expression changes were detected in the unmethylated SGC7901, AGS, N87 and MKN45 cell lines. Re-expression of NKD2 was induced in BGC823, MGC803 and AGS cells. Increased expression of NKD2 was observed in N87 cells. No NKD2 expression changes were demonstrated in the unmethylated SGC7901 and MKN45 cells. These results suggest that the expression levels of NKD1 and NKD2 are regulated by promoter region methylation in human gastric cancer cells.

### *NKD2* methylation is related to gastric cancer progression and metastasis and may serve as a poor prognostic predictor

**Figure 2:**
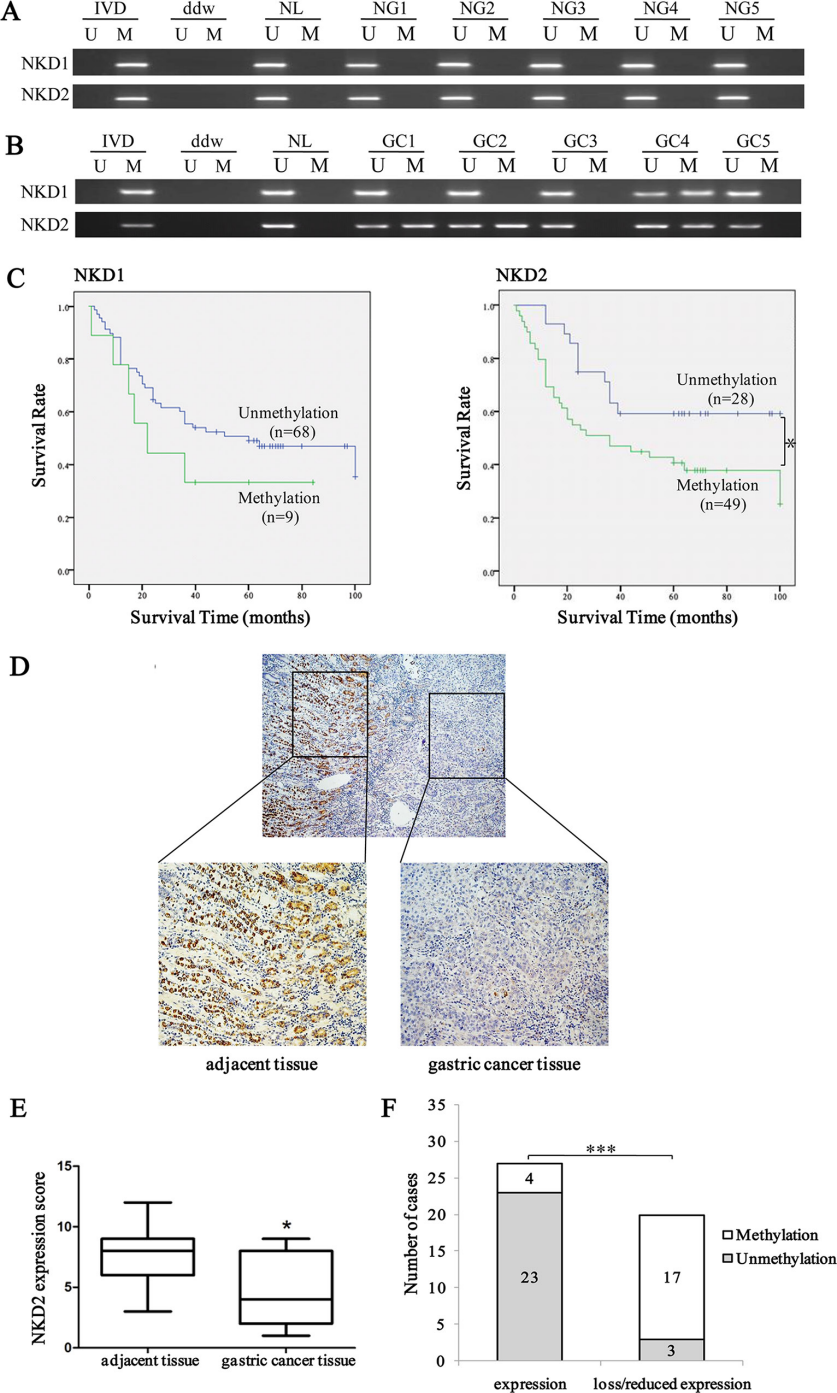
Methylation and expression status of *NKD2* in primary gastric cancer and their prognostic value **A.** MSP results of *NKD1* and *NKD2* in normal gastric mucosa. NG: normal gastric mucosa. **B.** Representative results of MSP for *NKD1* and *NKD2* in primary gastric cancer samples. GC: primary gastric cancer samples. **C.** The survival of gastric cancer patients in methylated and unmethylated *NKD1* and *NKD2* cases. Methylation of *NKD2* was associated with poor prognosis (*P < 0.05). **D.** Representative IHC results show NKD2 expression in gastric cancer and adjacent tissue samples (upper: ×40; lower: ×200). **E.** NKD2 expression scores are shown as box plots, horizontal lines represent the median score; the bottom and top of the boxes represent the 25^th^ and 75^th^ percentiles, respectively; vertical bars represent the range of data. Expression of NKD2 was significantly different between adjacent tissue and gastric cancer tissue in 47-matched primary gastric cancer samples. *P < 0.05. **F.** The expression of NKD2 and DNA methylation status is shown as a bar diagram. Reduced expression of NKD2 was significantly associated with promoter region hypermethylation. ***P < 0.001.

Methylation of *NKD1* and *NKD2* was examined in 196 cases of primary gastric cancer and 28 cases of normal gastric mucosa. No methylation was found in these genes in normal human gastric mucosa (Figure 2A). *NKD1* was methylated in 11.7% (23/196) of human gastric cancer, and methylation of *NKD1* was not associated with cell differentiation, TNM stage, distant metastasis, age, gender, tumor size, location and vessel invasion (all *P* > 0.05, Table 1). As shown in Figure 2C, no significant difference was found between the *NKD1* unmethylation and methylation groups for overall survival time (log-rank, *P* = 0.26). The above results demonstrate that *NKD1* methylation may not play an important role in gastric carcinogenesis and progression. *NKD2* was methylated in 53.1% (104/196) of human primary gastric cancer samples (Figure 2B), and methylation of *NKD2* was significantly associated with the degree of cell differentiation, TNM stage and distant metastasis (all *P* < 0.05, Table 2). Meanwhile no association was found between *NKD2* methylation and age, gender, tumor size, location and vessel invasion. As shown in Figure 2C, the overall survival was longer in the *NKD2* unmethylation group compared to the *NKD2* methylation group (*P* < 0.05), suggesting that *NKD2* methylation is related to tumor malignancy, progression and metastasis, and it may serve as a poor prognostic predictor in human gastric cancer. While, the COX regression analysis indicated that *NKD2* methylation is not an independent prognostic factor in gastric cancer.

**Table 1:** Clinical factors and *NKD1* methylation in 196 cases of gastric cancer samples

Clinical factor	No.	*NKD1* methylation status		P*value
		Methylated *n* = 23 (11.7%)	Unmethylated *n* = 173 (87.2%)	
***Age (year)***				
<50	40	3	37	*P* = 0.5109
≥50	156	20	136	
***Gender***				
Male	138	16	122	*P* = 0.9249
Female	58	7	51	
***Tumor Size (cm)***				
≤5	110	13	97	*P* = 0.9672
>5	86	10	76	
***Tumor Location***				
Upper	42	3	39	*P* = 0.3624
Middle	55	9	46	
Lower	99	11	88	
***Differentiation***				
Well	10	0	10	*P* = 0.1596
Moderate	57	4	53	
Poor	129	19	110	
***TNM Stage***				
I	17	1	16	*P* = 0.5921
II	51	8	43	
III	101	10	91	
IV	27	4	23	
***Distant metastasis***				
M0	174	20	154	*P* = 0.9542
M1	22	3	19	
***Vessel Invasion***				
Negative	115	14	101	*P* = 0.9982
Positive	81	9	72	

* *P* values are obtained from chi-square test, significant difference, *P* < 0.05

**Table 2:** Clinical factors and *NKD2* methylation in 196 cases of gastric cancer samples

Clinical factor	No.	*NKD2* methylation status		P*value
		Methylated *n* = 104 (53.1%)	Unmethylated *n* = 92 (46.9%)	
***Age (year)***				
<50	40	22	18	*P* = 0.7830
≥50	156	82	74	
***Gender***				
Male	138	71	67	*P* = 0.4855
Female	58	33	25	
***Tumor Size (cm)***				
≤5	110	63	47	*P* = 0.1815
>5	86	41	45	
***Tumor Location***				
Upper	42	22	20	*P* = 0.7396
Middle	55	27	28	
Lower	99	55	44	
***Differentiation***				
Well	10	1	9	*P* = 0.0146 < 0.05
Moderate	57	34	23	
Poor	129	69	60	
***TNM Stage***				
I	17	13	4	*P* = 0.0297 < 0.05
II	51	24	27	
III	101	48	53	
IV	27	19	8	
***Distant metastasis***				
M0	174	88	86	*P* = 0.0498 < 0.05
M1	22	16	6	
***Vessel Invasion***				
Negative	115	56	59	*P* = 0.1445
Positive	81	48	33	

* *P* values are obtained from chi-square test, significant difference, *P* < 0.05

As *NKD1* methylation did not appear to be a major event in human gastric cancer, we mainly focused on the mechanisms of NKD2 in gastric carcinogenesis. The expression of NKD2 was evaluated by IHC in 47 cases of available matched gastric cancer and adjacent tissue samples. The expression of NKD2 was apparently reduced in 20 cases of gastric cancer tissue samples compared to adjacent tissue samples (Figure 2D and 2E). In 20 cases of loss/reduced expression of NKD2 cancer samples, 17 cases were methylated. In 27 cases of NKD2 expressed cancer samples, 4 cases were methylated. Loss/reduced expression of NKD2 was related to promoter region hypermethylation (*P* < 0.001, Figure 2F). These results indicate that NKD2 expression may be regulated by promoter region methylation in human primary gastric cancer.

### Restoration of NKD2 expression suppresses cell growth in BGC823 and MGC803 cells

**Figure 3:**
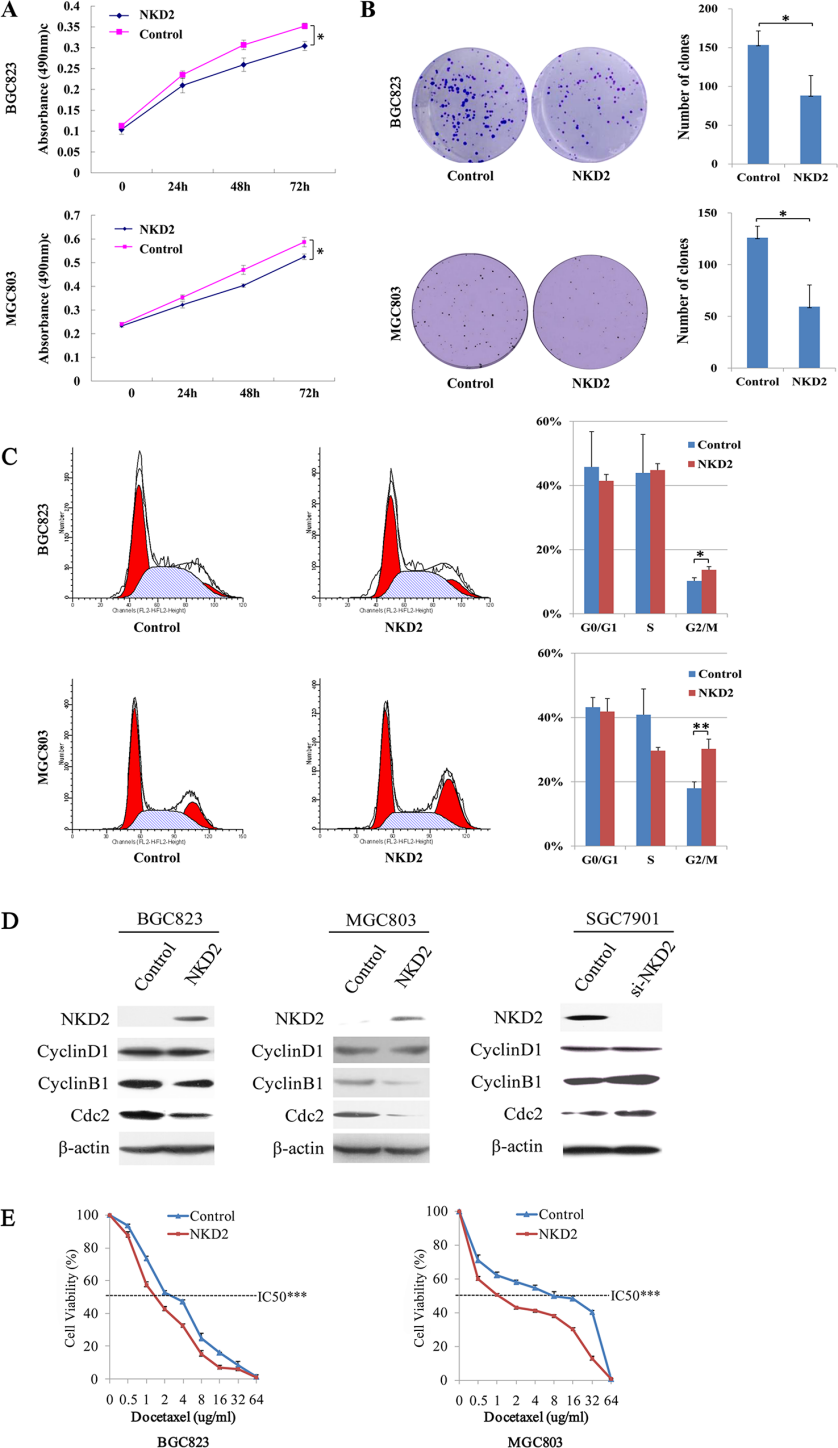
NKD2 induced G2/M phase arrest and sensitized BGC823 and MGC803 cells to docetaxel **A.** Growth curves represent the cell viability analyzed by the MTT assay in NKD2 re-expressed and unexpressed BGC823 and MGC803 cells. Each experiment was repeated in triplicate. *P < 0.05. **B.** Colony formation results show that colony number was reduced by re-expression of NKD2 in BGC823 and MGC803 cells. Each experiment was repeated in triplicate. Average number of tumor clones is represented by bar diagram. *P < 0.05. **C.** Cell phase distribution in NKD2 unexpressed and re-expressed BGC823 and MGC803 cells. The ratio is presented by bar diagram. Each experiment was repeated for three times. *P < 0.05, **P < 0.01. **D.** The expression of NKD2, CyclinD1, CyclinB1 and Cdc2 was detected by western blot in NKD2 unexpressed and re-expressed BGC823 and MGC803 cells. Knockdown of NKD2 in SGC7901 cells was used to validate the results. β-actin: internal control. **E.** The cell viability assay showed the effect of NKD2 on the sensitivity of BGC823 and MGC803 cells to docetaxel. ***P < 0.001.

The effect of NKD2 on cell proliferation was evaluated by the MTT assay in BGC823 and MGC803 cells. The OD value was 0.352 ± 0.007 vs. 0.304 ± 0.011 (*P* < 0.05) in BGC823 cells and 0.587 ± 0.020 vs 0.525 ± 0.012 (*P* < 0.05) in MGC803 cells before and after restoration of NKD2 expression (Figure 3A).

The colony formation assay was performed in BGC823 and MGC803 cells. The clone number was 153.3 ± 18.1 vs. 88.3 ± 25.8 (*P* < 0.05) in BGC823 cells and 126.0 ± 11.1 vs. 59.3 ± 21.0 (*P* < 0.05) in MGC803 cells before and after restoration of NKD2 expression (Figure 3B).

### NKD2 induced G2/M phase arrest and sensitized BGC823 and MGC803 cells to docetaxel

Flow cytometry was employed to analyze the effects of NKD2 on the cell cycle. As shown in Figure 3C, the distribution of cell phase in the NKD2 unexpressed and re-expressed BGC823 cell line was 45.81 ± 0.11% vs. 41.48 ± 0.02% in G0/G1 phase, 43.94 ± 0.12% vs. 44.82 ± 0.02% in S phase, and 10.25 ± 0.01% vs. 13.71 ± 0.01% in G2/M phase. The G2/M phase was significantly different before and after re-expression of NKD2 (*P* < 0.05). In MGC803 cells, the cell phase distribution was 43.20 ± 0.03% vs. 41.86 ± 0.04% in G0/G1 phase, 40.88 ± 0.08% vs. 29.68 ± 0.01% in S phase, and 17.98 ± 0.02% vs. 30.26 ± 0.03% in G2/M phase before and after restoration of NKD2 expression. The G2/M phase was significantly different before and after re-expression of NKD2 in MGC803 cells (*P* < 0.01).

The role of NKD2 in cell cycle regulation was further validated by the detection of G2/M phase related proteins. The expression of CyclinB1 and Cdc2 were dramatically reduced after re-expression of NKD2 in BGC823 and MGC803 cells, while the expression of CyclinD1 did not change before and after re-expression of NKD2. The effect of NKD2 on G2/M phase was further confirmed by the knocking down of NKD2 in SGC7901 cells. The expression levels of CyclinB1 and Cdc2 were increased after knockdown of NKD2 in SGC7901 cells (Figure 3D).

Docetaxel, a microtubule inhibitor, exerts its effects on the G2/M checkpoint. To determine whether NKD2 is involved in docetaxel sensitivity, we examined the cell viability of NKD2 unexpressed and re-expressed BGC823 and MGC803 cells after docetaxel treatment. The correlation of cell viability and docetaxel concentration was shown in Figure 3E. The IC_50_ of docetaxel was 3.019 ± 0.093 vs. 1.761 ± 0.054 ug/ml (*P* < 0.001) in BGC823 cells and 5.099 ± 0.339 vs. 1.433 ± 0.068 ug/ml (*P* < 0.001) in MGC803 cells before and after restoration of NKD2 expression. These results indicate that NKD2 functions as a mitotic inhibitor in human gastric cancer cells and sensitizes these cells to docetaxel.

### Restoration of NKD2 expression inhibits cell invasion, migration and wound healing ability

**Figure 4:**
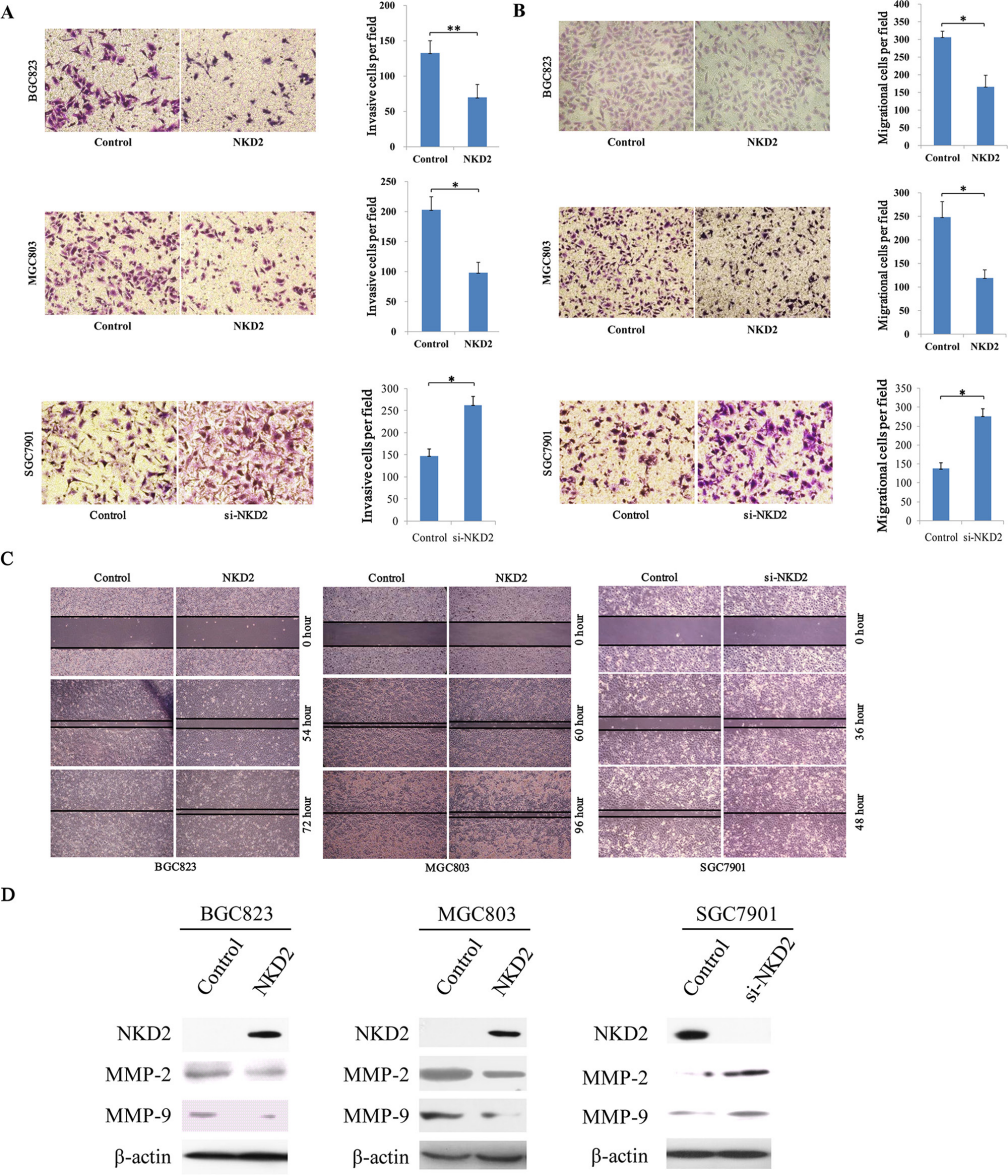
NKD2 inhibites cell invasion, migration and wound healing ability **A.** Cell invasion in NKD2 unexpressed and expressed BGC823 and MGC803 cells, as well as cell invasion before and after knocking down NKD2 in SGC7901 cells. The invasive cell number is presented by bar diagram. Each experiment was repeated for three times. *P < 0.05, **P < 0.01. **B.** Cell migration in NKD2 unexpressed and expressed BGC823 and MGC803 cells, as well as cell migration before and after knocking down NKD2 in SGC7901 cells. The migrational cell number is presented by bar diagram. Each experiment was repeated for three times. *P < 0.05. **C.** Wound healing results in NKD2 unexpressed and expressed BGC823 and MGC803 cells, as well as the wound healing results before and after knockdown of NKD2 in SGC7901 cells. Each experiment was repeated for three times. **D.** The expression levels of NKD2, MMP-2 and MMP-9 were detected by western blot in NKD2 unexpressed and expressed BGC823 and MGC803 cells. Knockdown of NKD2 in SGC7901 cells was performed to validate these results.

The transwell assay was employed to evaluate the effect of NKD2 on cell invasion in BGC823 and MGC803 cells. The number of invasive cells for each high power field under the microscope was 132.7 ± 17.4 vs. 70.0 ± 18.1 in BGC823 cells and 203.0 ± 21.8 vs. 98.3 ± 17.2 in MGC803 cells before and after restoration of NKD2 expression. The cell number was significantly different before and after re-expression of NKD2 in BGC823 and MGC803 cells (*P* < 0.01, *P* < 0.05, respectively). The effect of NKD2 on cell invasion was further validated by knocking down NKD2 in SGC7901 cells. The number of invasive cells was 148.0 ± 15.5 vs. 263.3 ± 19.4 before and after knockdown NKD2 in SGC7901 cells in each high power field under the microscope (*P* < 0.05, Figure 4A).

Next, the transwell assay in the absence of ECM gel (extracellular matrix gel) coating was employed to explore the effect of NKD2 on cell migration in BGC823 and MGC803 cells. The number of migrated cells for each high power field under the microscope was 306.3 ± 17.0 vs. 166.0 ± 32.5 in BGC823 cells and 248.3 ± 32.9 vs. 119.0 ± 17.3 in MGC803 cells before and after restoration of NKD2 expression. The cell number was significantly different before and after re-expression of NKD2 (all *P* < 0.05). The effect of NKD2 on cell migration was further validated by knocking down NKD2 in SGC7901 cells. The number of migrational cells for each high power field under the microscope was 137.3 ± 15.9 vs. 276.7 ± 19.6 before and after knockdown NKD2 in SGC7901 cells (*P* < 0.05, Figure 4B).

The wound healing assay was also employed to measure the cell migration ability. As shown in Figure 4C, the migration of BGC823 and MGC803 cells was apparently suppressed after re-expression of NKD2. And the migration ability of SGC7901 cells was promoted by knocking down NKD2. To further validate the effect of NKD2, the expression levels of MMP-2 and MMP-9 were detected by western blot. As shown in Figure 4D, the expression levels of MMP-2 and MMP-9 were reduced after re-expression of NKD2 in BGC823 and MGC803 cells. The inhibitory role of NKD2 in MMP-2 and MMP-9 expression was further validated by the knocking down of NKD2 in SGC7901 cells. These results demonstrate that NKD2 suppresses gastric cancer cell invasion and migration.

### NKD2 suppresses gastric cancer cell growth in xenograft mice

**Figure 5:**
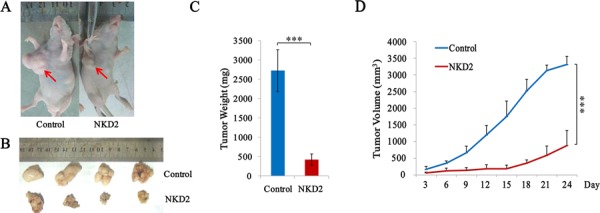
NKD2 suppresses gastric cancer cell growth in xenograft mice **A.** Representative burdened nude mice in NKD2 expressed and unexpressed BGC823 cells. Red arrows show position of subcutaneous tumors. **B.** Representative xenografts in NKD2 re-expressed and unexpressed BGC823 cells. **C.** Tumor weight in nude mice at the 24th day after inoculation of NKD2 unexpressed and expressed BGC823 cells. Bars: mean of 4 mice. ***P < 0.001. **D.** The tumor volumes for NKD2 unexpressed and expressed BGC823 cell xenografts. Points: mean of 4 mice. ***P < 0.001.

To further validate the effects of NKD2 in gastric cancer, NKD2 unexpressed and re-expressed BGC823 cell xenograft mouse models were employed (Figure 5A and 5B). The xenograft tumor weight was 2727.0 ± 541.6 mg in NKD2 unexpressed BGC823 cell tumors and 427.6 ± 143.0 mg in NKD2 re-expressed BGC823 cell tumors. The tumor weights were significantly different (*P* < 0.001, Figure 5C). The tumor volume was 3315.4 ± 249.9 mm^3^ in NKD2 unexpressed BGC823 cell xenografts and 890.2 ± 448.3 mm^3^ in NKD2 re-expressed BGC823 cell xenografts. The tumor volumes were smaller in NKD2 re-expressed BGC823 cells compared to NKD2 unexpressed BGC823 cells (*P* < 0.001, Figure 5D). These results suggest that NKD2 suppresses gastric cancer cell growth *in vivo*.

### NKD2 suppresses gastric cancer cell invasion and migration by down-regulating SOX18 expression

**Figure 6:**
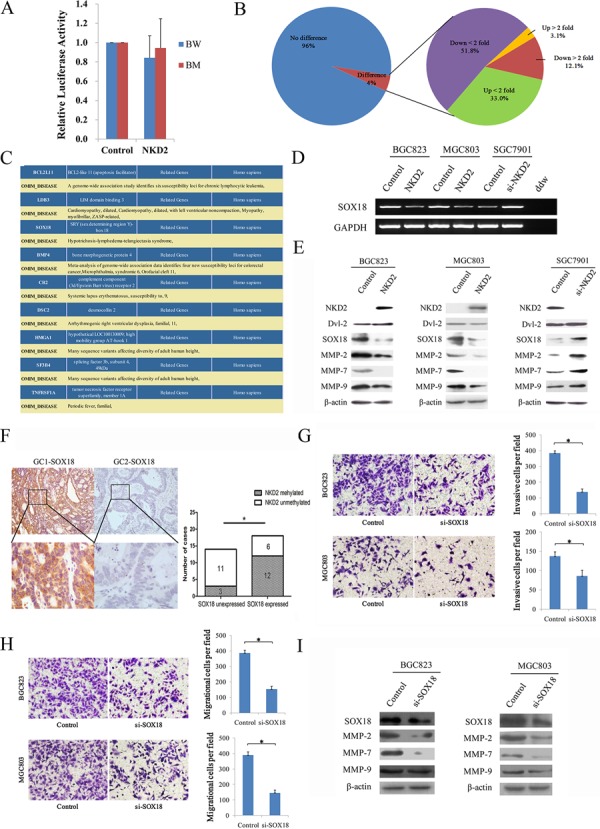
NKD2 suppresses SOX18 and MMP-2,7,9 expression in gastric cancer **A.** Results of TCF/LEF luciferase reporter assay. Relative luciferase activity (a ratio of firefly luciferase to renilla luciferase) was not changed after co-transfection of NKD2 with wild type or mutant type β-catenin in BGC823 cells (*P* > 0.05). The experiment was repeated three times. **B.** Gene expression array shows differentially expressed genes in NKD2 re-expressed and unexpressed BGC823 cells. The percentage of up-regulated or down-regulated genes are shown in the pie chart. Angle of the sector: the percentage of each category. **C.** *BCL2L11, LDB3, SOX18, BMP4, CR2, DSC2, HMGA1, SF3B4* and *TNFRSF1A* genes were associated with disease according to DAVID software (http://david.abcc.ncifcrf.gov/home.jsp). **D.** The expression of SOX18 was detected by semi-quantitative RT-PCR in NKD2 unexpressed and re-expressed BGC823 and MGC803 cells. The results were further validated by knockdown of NKD2 in SGC7901 cells. **E.** The expression levels of NKD2, Dvl-2, SOX18 and MMP-2,7,9 were detected by western blot in NKD2 unexpressed and re-expressed BGC823 and MGC803 cells. The results were validated by knocking down NKD2 in SGC7901 cells. **F.** Representative IHC results show SOX18 expression in primary gastric cancer (upper: ×100; lower: ×400). NKD2 is methylated in GC1 and unmethylated in GC2. The correlation of SOX18 expression and NKD2 methylation status is shown as a bar diagram. The expression of SOX18 is associated with NKD2 methylation significantly. GC: primary gastric cancer samples. *P < 0.05. **G.** Cell invasion in BGC823 and MGC803 cells before and after knockdown SOX18. The invasive cell number is presented by bar diagram. Each experiment was repeated for three times. *P < 0.05. **H.** Cell migration in BGC823 and MGC803 cells before and after knockdown SOX18. The migrational cell number is presented by bar diagram. Each experiment was repeated for three times. *P < 0.05. **I.** The expression levels of SOX18 and MMP-2,7,9 were detected by western blot before and after knockdown SOX18 in BGC823 and MGC803 cells.

NKD2 has been shown to negatively regulate canonical Wnt signaling through an interaction with Dishevelled. To explore the mechanism of NKD2 in gastric cancer, a Topflash reporter and TCF/LEF luciferase reporter assay were used in BGC823 cells. As shown in Figure 6A, relative luciferase activity was not changed after co-transfection of NKD2 with wild type or mutant type of β-catenin in BGC823 cells (*P* > 0.05) These data suggest that the activity of the TCF/LEF reporter was not affected by NKD2. The expression of Dvl-2, the NKD2 binding protein, was not changed before and after re-expression of NKD2 in BGC823 and MGC803 cells. No obvious Dvl-2 expression changes were found in SGC7901 cells before and after the knockdown of NKD2 (Figure 6E). These results demonstrate that NKD2 does not directly inhibit canonical Wnt signaling.

To further understand the mechanisms of NKD2 in gastric cancer progression, a gene expression array was employed. The gene expression profile was analyzed in NKD2 re-expressed and unexpressed BGC823 cells. Among a total of 31741 genes, expression changes were found in 1376 genes (*P* < 0.05). Among these genes, 43 genes were up-regulated and 167 genes were down-regulated more than 2-fold (Figure 6B). DAVID software (http://david.abcc.ncifcrf.gov/home.jsp) was employed to obtain the disease related genes. Among these apparently changed genes, *BCL2L11, LDB3, SOX18, BMP4, CR2, DSC2, HMGA1, SF3B4* and *TNFRSF1A* were regarded as disease-related genes according to DAVID (Figure 6C). SOX18 was validated to be down-regulated by semi-quantitative RT-PCR in NKD2 re-expressed BCG823 and MGC803 cells, and up-regulation of SOX18 was verified by the knocking down NKD2 in SGC7901 cells (Figure 6D). These results suggest that SOX18 is down-regulated by NKD2 in human gastric cancer.

In human umbilical vein endothelial cells (HUVEC), metalloproteinase-7 (MMP-7) is up-regulated by SOX18, and confirmed that MMP-7 is a direct target of SOX18 [27]. In our study, the expression levels of SOX18 and MMP-2, 7, 9 were reduced in NKD2 re-expressed BGC823 and MGC803 cells. Up-regulation of SOX18 and MMP-2,7,9 was found after knockdown NKD2 in SGC7901 cells (Figure 6E). To further validate SOX18 is regulated by NKD2, we analyzed the association between SOX18 overexpression and NKD2 methylation in human primary gastric cancer. The expression of SOX18 was examined by IHC in 32 cases primary gastric cancer samples. The expression level of SOX18 is associated with *NKD2* methylation significantly (*P* < 0.05, Figure 6F). The results suggest that the expression of SOX18 is regulated by NKD2 in human primary gastric cancer.

To further validate that cell invasion and metastasis is induced by SOX18, siRNA knockdown technique was performed. As shown in Figure 6G and 6H, the number of invasive cells is 384.3 ± 14.8 vs. 138.3 ± 18.6 in BGC823 cells and 136.7 ± 11.6 vs. 86.0 ± 14.5 in MGC803 cells before and after knockdown SOX18 in each high power field (all *P* < 0.05). And the number of migrational cells is 386.7 ± 18.0 vs. 153.0 ± 18.7 in BGC823 cells and 389.0 ± 21.7 vs. 146.0 ± 17.3 in MGC803 cells before and after knockdown SOX18 in each high power field (all *P* < 0.05). The expression levels of MMP-2, 7, 9 were reduced after knockdown SOX18 in BGC823 and MGC803 cells (Figure 6I). Above results suggest that NKD2 impedes cell invasion and metastasis by inhibiting SOX18 in gastric cancer.

## DISCUSSION

In this study, we detected the methylation status of *NKD1* and *NKD2* in human gastric cancer cell lines and primary cancer tissue samples. The expression levels of NKD1 and NKD2 were regulated by promoter region methylation. *NKD1* was infrequently methylated in primary gastric cancer, while *NKD2* was frequently methylated. Methylation of *NKD2* was related to cell differentiation, TNM stage, distant metastasis and overall survival, but no association was found between *NKD1* methylation and any of the analyzed clinical factors. Therefore, we mainly focused our study on NKD2 in gastric cancer. NKD2 inhibited cell proliferation, colony formation, and induced G2/M phase arrest. Re-expression of NKD2 sensitized gastric cancer cells to docetaxel. These results suggest that *NKD2* methylation is involved in gastric cancer prognosis and metastasis. It may serve as a gastric cancer diagnostic, prognostic and chemo-sensitive marker.

The role of NKD2 in gastric cancer progression and metastasis was further studied both *in vitro* and *in vivo*. The growth of gastric cancer xenografts was suppressed by NKD2. Cell invasion and migration was impeded by NKD2 in BGC823 and MGC803 cells. The mechanisms of NKD2 in gastric cancer cell invasion and migration were further investigated by detecting the expression of MMP-2 and MMP-9. The effect of NKD2 on the inhibition of MMP-2 and MMP-9 expression levels was revealed by re-expression of NKD2 in unexpressed cells, and this was further validated by knocking down NKD2 in highly expressed cells. By analyzing the gene expression profiles in NKD2 re-expressed and unexpressed BGC823 cells, 43 up-regulated and 167 down-regulated genes were found to be changed more than 2 fold. Among these genes, 9 were found to be related to disease according to DAVID software. Of these 9 genes, SOX18 has been reported to be related to cancer progression, invasion and metastasis in mouse and human cancers [28–30]. Down-regulation of SOX18 by NKD2 was validated by RT-PCR and western blot in gastric cancer cells. The expression of SOX18 is associated with NKD2 methylation significantly in primary gastric cancer. MMP-7 has been reported to be directly up-regulated by SOX18 [27]. We detected the expression of MMP-2,7,9 upon re-expression of NKD2 in unexpressed cells and knocking down of NKD2 in highly expressed cells. Down-regulation of SOX18 and MMP-2,7,9 by NKD2 was verified by both of these approaches. Further study found that SOX18 promotes cell invasion and metastasis by down regulating MMP-2,7,9 in gastric cancer cells. All of the above results suggest that NKD2 suppresses gastric cancer progression and metastasis by down-regulation of SOX18.

In conclusion, *NKD2* is frequently methylated in human gastric cancer, and methylation of *NKD2* is involved in gastric carcinogenesis. *NKD2* methylation may serve as a gastric cancer diagnostic, prognostic and chemo-sensitive marker. NKD2 suppresses gastric cancer metastasis by down-regulating SOX18 and its downstream genes.

## MATERIALS AND METHODS

### Human tissue samples and cell lines

Primary human gastric cancer cases (196) were collected from the Chinese PLA General Hospital in Beijing and the Ruijin Hospital in Shanghai. The median age of the cancer patients was 62 years old (range 20–87), and the ratio of males/females was 2.4:1. All cancer samples were classified according to TNM staging (AJCC 2010), including 17 cases of stage I, 51 cases of stage II, 101 cases of stage III and 27 cases of stage IV. The 5-years survival follow-up data were available for 77 cases. Twenty eight cases of normal gastric mucosa from patients without cancer were collected by endoscopy biopsy at the Chinese PLA General Hospital. All samples were collected following the guidelines approved by the institutional review board of the Chinese PLA General and Ruijin Hospitals with written informed consent from patients.

Six gastric cancer cell lines (SGC7901, MGC803, BGC823, AGS, N87, and MKN45) were previously established from primary gastric cancer and maintained in 90% RPMI 1640 (Invitrogen, Carlsbad, CA) supplemented with 10% fetal bovine serum.

### 5-aza-2′-deoxycytidine treatment

Gastric cancer cell lines were split to a low density (30% confluence) 12 hours before treatment. Cells were treated with 5-aza-2′-deoxycytidine (DAC) (Sigma, St. Louis, MO) at a concentration of 2 μM. Growth medium conditioned with DAC at a concentration of 2 μM was exchanged every 24 hours for a total of 96 hours of treatment.

### RNA isolation and semi-quantitative RT-PCR

Total RNA was isolated by Trizol reagent (Life Technologies, Gaithersburg, MD). First strand cDNA was synthesized according to the manufacturer's instructions (Invitrogen, Carlsbad, CA). PCR primers for NKD1 and NKD2 are listed in Supplementary Table S1. The primer sets for NKD1 and NKD2 were designed to span intronic sequences between adjacent exons in order to control for genomic DNA contamination. RT-PCR was amplified for 33 cycles. GAPDH was used as an internal control.

### Bisulfite modification, methylation-specific PCR (MSP) and bisulfite sequencing

DNA was prepared by the proteinase K method. Bisulfite treatment was carried out as previously described [31]. MSP primers were designed according to genomic sequences around transcription start sites (TSS) and synthesized to detect unmethylated (U) and methylated (M) alleles. Bisulfite sequencing (BSSQ) was performed as previously described [32]. BSSQ products were amplified by primers flanking the targeted regions including MSP products. All primers are listed in Supplementary Table S1.

### Immunohistochemistry

Immunohistochemistry (IHC) was performed in primary gastric cancer samples and paired adjacent tissue samples. The NKD2 antibody was diluted 1:500 (Novus Biology, CO, USA) and the SOX18 antibody was diluted 1:1000(LifeSpan BioSciences, Inc, WA, USA). The staining intensity and extent of the staining area were scored using the German semi-quantitative scoring system as previously described [32, 33].

### Construction of NKD2 expression vector and transfection assay

Full-length *NKD2* cDNA (GenBank accession number NM_033120) was cloned into the pCMV6 vector. Transient transfection was performed using Lipofectamine 2000 (Intrivogen, Carlsbad, CA) or FuGENE HD (Roche Applied Science, Indianapolis, IN) according to the manufacturer's instructions.

### Cell viability detection

Cells were plated into 96-well plates at 2 × 10^3^ cells/well, and the cell viability was measured by the MTT assay (KeyGEN Biotech, Nanjing, China) at 0, 24, 48 and 72 h. Absorbance was measured on a microplate reader (Thermo Multiskan MK3, MA, USA) at a wavelength of 490 nm.

The sensitivity of gastric cancer cells to docetaxel was analyzed by detecting the cell viability in NKD2 unexpressed or re-expressed cells treated by docetaxel at 0, 0.5, 1, 2, 4, 8, 16, 32 and 64 ug/ml for 48 hours. The IC_50_ was defined as the concentration required for 50% inhibition of cell growth. Absorbance was measured as specified above. The percentage of viable cells was calculated as follows: (%) = [A490(treated)–A490(blank)]/[A490(control)–A490(blank)] × 100%.

### Colony formation assay

NKD2 unexpressed and re-expressed BGC823 and MGC803 cells were seeded at 500 cells/well in 6-well plates in triplicate. Growth medium conditioned with G418 (Invitrogen, Carlsbad, CA) at 450 μg/ml was exchanged every 24 hours. Clones were counted 14 days after being fixed with 75% ethanol and stained with 0.2% crystal violet (Beyotime, Nanjing, China) for visualization and counting.

### Flow cytometry

Cells were starved 12 hours for synchronization, and the cells were re-stimulated with 10% FBS for 24 hours. Cells were fixed with 70% ethanol and treated using the Cell Cycle Detection Kit (KeyGenBiotech, Nanjing, China). The cells were then sorted by a FACS Caliber flow cytometer (BD Biosciences, Mansfield, CA). The cell phase distribution was analyzed by the Modfit software (Verity Software House, ME, USA).

### Transwell assay

Cells were suspended in serum-free medium. Cells (2 × 10^4^) were placed into the upper chamber of an 8 μm pore size Transwell apparatus (Corning, NY, USA) and incubated for 18 hours. Cells that migrated to the lower surface of the membrane were stained with crystal violet and counted in three independent high-power fields (×200). For invasion analysis, cells (2 × 10^4^) were seeded into the upper chamber of a transwell apparatus coated with Matrigel (BD Biosciences, San Jose, CA) and incubated for 36 hours. Cells that invaded into the lower membrane surface were stained with crystal violet and counted in three independent high-power fields (×200).

### Wound healing assay

Creation of a linear scratch wound was performed using a pipette tip in a confluent monolayer of cells in 6-well plates. Medium without FBS was used in order to inhibit cell proliferation.

### siRNA knockdown technique

Selected siRNAs targeting NKD2, siRNAs targeting SOX18 and the RNAi negative control duplex were used in this study. The sequences of the siRNAs are listed in Supplementary Table S1. The RNAi oligonucleotide and RNAi negative control duplex were transfected into NKD2 highly expressing SGC7901 cells or SOX18 expressing BGC823 cells and MGC803 cells.

### Luciferase reporter assay

Top flash reporter and TCF/LEF reporter luciferase reporter assay was employed in BGC823 cells as previously described [34]. Relative luciferase activity (a ratio of firefly luciferase to renilla luciferase) after co-transfection of NKD2 with wild type or mutant type of β-catenin in BGC823 cells was measured by the Dual Luciferase Reporter Assay system (Promega, Shanghai, China).

### Gene expression array

Total RNA was isolated from NKD2 re-expressed and unexpressed BGC823 cells by Trizol reagent (Life Technologies, Gaithersburg, MD) as previously described. The RNA samples were labeled with Cy5/Cy3 and hybridized to the Human Whole Genome One Array (Phalanx Biotech Group, CA, USA). The hybridized chips were scanned by an Axon 4000 scanner (Molecular Devices, CA, USA). Spot quantification was performed using the Genepix 4.1 software (Axon Instruments, CA, USA). Differentially expressed genes with fold changes >2 were further analyzed by DAVID bioinformatics (LIB, SAIC-Frederick, Inc., Frederick, MD).

### Western blot

Protein from gastric cancer cells was collected 48 hours after transfection and western blot was performed as described previously [35]. Antibodies were diluted according to manufacturer's instructions. The primary antibodies were as follows: NKD2, Dvl-2 (CST, MA, USA), MMP-2, MMP-9, CyclinD1, CyclinB1, Cdc2, MMP-7 (Bioworld Tech, MN, USA), SOX18 (Abcam, MA, USA), and β-actin (Beyotime Biotech., China).

### NKD2 re-expressed and unexpressed BGC823 cell xenograft mouse model

NKD2 re-expressed or unexpressed BGC823 cells (2 × 10^6^) were suspended in 0.1 ml PBS and injected subcutaneously into the right armpit of each 4-week-old female Balb/c nude mouse (6 per group). The diameter of the tumors was measured every 3 days. Tumor volume (mm^3^) was estimated by the following formula: tumor volume = (length) × (width)^2^/2. Mice were sacrificed at 24 days, and tumor size and weight were measured after dissection. All procedures were approved by the Animal Ethics Committee of the Chinese PLA General Hospital.

### Statistical analysis

SPSS 17.0 software (IBM, NY, USA) was used for data analysis. The student's t test, the Pearson chi-squared test, the Fisher's exact test and the log-rank test (Kaplan-Meier) and COX regression analysis were used in this study. *P* < 0.05 was regarded as a statistically significant difference.

## SUPPLEMENTARY TABLE


