# Supplementary material for: Silencing NKD2 by promoter region hypermethylation promotes gastric cancer invasion and metastasis by up-regulating SOX18 in human gastric cancer
Source: Oncotarget. 2015 Sep 14;6(32):33470–85. doi: 10.18632/oncotarget.5272 (PMC4741779; doi:10.18632/oncotarget.5272)
Supplement: Supplementary file 1 [file oncotarget-06-33470-s001.pdf]

SUPPLEMENTARY TABLE

Supplementary Table S1: Primer Sequences

**RT-PCR primers**
NKD1-F: 5′-AACCACTACTTAGATCTCGCCG-3′
NKD1-R: 5′-GAGCCGTTGCTGGAGCTCTG-3′
NKD2-F: 5′-ACAGGAGGTTGTCTGCACACG-3′
NKD2-R: 5′-GACTTGAGGAACTGCTTCTCCG-3′
GAPDH-F: 5′-GACCACAGTCCATGCCATCAC-3′
GAPDH-R: 5′-GTCCACCACCCTGTTGCTGTA-3′
**MSP primers**
NKD1-ME-F: 5′-GTTCGGCGTTTTCGGGCGTTAGTC-3′
NKD1-UN-F: 5′-GGGTTTGGTGTTTTTGGGTGTTAGTT-3′
NKD1-ME-R: 5′-AAATTTCCCCATACTAAAACTACGACG-3′
NKD1-UN-R: 5′-TAAAATTTCCCCATACTAAAACTACAACA-3′
NKD2-ME-F: 5′-GATCGTAGGGGATAGTTTCGTGGC-3′
NKD2-UN-F: 5′-TGGATTGTAGGGGATAGTTTTGTGGT-3′
NKD2-ME-R: 5′-AAAACAACTCTAACACCGCTCCCCG-3′
NKD2-UN-R: 5′-CAAAAACAACTCTAACACCACTCCCCA-3′
**BSSQ primers**
NKD1-F: 5′-ATGYGTYGTATYGGTTAATGGG-3′
NKD1-R: 5′-AACRAAAACACTAACCCRACTTA-3′
NKD2-F: 5′-GGGGATATAGYGAAGGYGTAG-3′
NKD2-R: 5′-TACRACCATAACRACCCACRTC-3′
**siRNA duplex sequence**
NKD2-F: 5′-GGGAUUGAGAACUACACGUTT-3′
NKD2-R: 5′-ACGUGUAGUUCUCAAUCCCTT-3′
SOX18-F: 5′- CUCUCUCAUACGCGUGUAUTT-3′
SOX18-R: 5′- AUACACGCGUAUGAGAGAGTT-3′
Negative Control-F: 5′-UUCUCCGAACGUGUCACGUTT-3′
Negative Control-R: 5′-ACGUGACACGUUCGGAGAATT-3′
